# Supplementary material for: Nutritional Quality and Safety of the Spirulina Dietary Supplements Sold on the Slovenian Market
Source: Foods. 2022 Mar 17;11(6):849. doi: 10.3390/foods11060849 (PMC8954120; doi:10.3390/foods11060849)
Supplement: Supplementary file 1 [file foods-11-00849-s001.zip › foods-1593300-supplementary.pdf]

Table S1. Amino acid composition of *Spirulina* supplements available on the Slovenian market (mg/g dwt).

Sample	ALA[1]	GLY[2]	VAL[3]	LEU[4]	ILE[5]	THR[6]	SER[7]	PRO[8]	ASP[9]	MET[10]	GLU[11]	PHE[12]	LYS[13]	HIS[14]	TYR[15]
S1	39.2	28.4	95.6	53.8	66.8	44.6	42.4	28.7	76.0	16.4	164	34.6	65.3	16.3	29.5
S2	38.0	27.8	91.7	54.4	65.2	43.9	41.5	28.4	74.0	18.0	167	34.5	36.3	17.2	29.1
S3	37.5	28.8	91.6	51.2	68.1	46.6	44.8	28.4	80.2	19.8	175	35.5	66.5	18.1	32.5
S4	42.0	30.7	98.6	57.6	70.0	47.5	43.9	31.9	79.5	14.7	158	36.8	56.9	18.9	32.1
S5	40.7	29.5	101	54.8	72.2	47.8	47.2	30.5	84.7	16.3	190	36.1	88.9	19.2	32.3
S6	38.2	31.9	85.8	49.8	48.0	44.3	37.8	34.9	74.9	11.6	116	37.6	126	19.9	28.3
S7	28.7	24.1	71.1	44.8	57.9	47.5	42.3	25.2	85.6	20.6	195	36.6	88.3	18.8	33.9
S8	44.8	33.3	104	58.7	75.0	54.8	52.5	31.3	92.5	19.5	220	40.3	77.5	19.6	37.0
S9	21.0	16.3	50.4	32.1	33.3	27.1	25.4	20.0	47.3	12.7	90.5	24.0	48.5	15.8	18.6
S10	42.5	32.1	102	53.5	73.9	54.8	52.5	30.8	91.9	18.6	199	39.1	76.6	19.2	36.6
S11	39.0	29.5	101	56.4	75.0	50.3	46.2	30.8	91.4	22.7	145	36.7	72.9	19.2	34.8
S12	46.1	33.1	109	58.4	78.9	49.1	41.5	31.3	88.5	21.4	187	35.6	68.5	18.3	36.4
S13	41.5	29.8	102	50.8	71.9	49.8	47.0	28.8	83.6	18.8	154	34.0	72.7	18.3	32.6
S14	47.3	32.2	116	54.8	79.4	45.3	38.1	30.7	80.6	19.3	151	34.0	70.7	18.8	34.5
S15	43.5	30.5	108	58.6	76.1	52.1	50.6	30.9	87.9	22.8	180	37.2	95.0	20.8	37.8
S16	38.5	29.8	91.5	51.2	68.0	45.0	36.9	27.5	83.9	20.7	162	36.0	75.0	19.0	37.4
S17	44.7	33.4	107	52.9	76.0	52.8	51.5	30.4	92.2	20.4	161	36.8	96.9	20.1	36.3
S18	32.6	25.4	67.5	45.0	46.6	36.4	32.7	24.9	63.4	15.9	122	32.0	77.9	17.7	25.8
S19	21.8	18.1	55.4	38.0	44.2	35.6	33.5	20.2	61.4	16.1	106	26.7	58.1	17.2	24.8
S20	33.1	27.3	78.1	51.9	60.9	45.8	42.5	26.0	78.9	19.3	140	34.1	61.5	19.2	32.1
S21	26.5	31.3	109	55.5	73.5	49.9	46.9	31.3	85.7	22.0	158	36.3	66.6	20.5	33.5
S22	20.8	15.6	50.8	30.0	37.0	24.7	21.8	17.3	41.5	12.5	72.5	21.1	30.3	16.3	17.5
S23	41.6	30.1	105	54.6	74.5	49.7	45.9	30.5	85.3	20.0	160	36.2	86.9	19.8	34.9
S24	41.3	29.1	106	57.5	73.1	48.3	46.2	30.6	82.8	20.6	155	33.8	78.2	21.2	32.4
S25	37.0	27.2	94.6	55.1	67.2	47.1	44.0	27.9	80.3	17.9	167	33.4	64.6	19.0	32.4
S26	38.5	29.3	94.9	56.5	67.9	50.3	47.6	33.4	83.4	21.5	146	34.7	92.4	20.8	33.3
S27	42.7	31.6	104	61.4	74.3	54.6	53.1	30.2	89.4	22.0	196	36.4	81.7	20.8	36.8
S28	38.4	29.3	102	60.4	75.7	53.6	51.3	29.8	90.4	22.8	209	37.2	97.6	22.2	38.4
S29	41.5	29.9	107	56.8	77.9	52.7	50.5	30.8	88.3	22.5	213	38.8	113	22.0	38.0
S30	36.4	27.2	96.0	56.7	69.2	50.4	48.0	28.5	85.3	19.6	148	35.2	93.8	20.4	34.8
S31	45.3	32.0	116	60.3	82.7	56.5	52.2	33.0	96.8	21.8	165	40.3	102	21.9	37.9
S32	35.4	25.7	92.5	53.2	66.2	45.5	42.1	27.8	76.1	19.9	127	32.2	64.0	19.0	30.8
S33	37.9	26.8	96.0	56.9	70.8	47.7	43.7	28.5	79.7	20.4	152	34.0	77.6	19.5	33.2
S34	36.7	26.3	93.1	54.9	68.4	44.3	42.1	27.8	76.5	20.2	138	32.2	66.7	18.3	30.5
S35	43.8	30.3	109	61.7	78.6	49.6	46.2	31.0	82.6	22.3	158	36.6	67.8	20.0	34.7
S36	49.3	34.0	123	67.4	84.4	58.9	58.2	34.6	101	23.2	197	39.1	93.0	21.8	38.4
S37	26.8	18.4	66.4	37.4	47.4	33.5	33.6	20.9	58.1	15.6	135	24.3	47.3	17.7	22.3
S38	43.7	30.1	113	63.0	80.7	55.5	53.2	31.2	94.3	23.3	202	38.3	97.6	21.8	38.4
S39	42.7	31.3	111	64.2	81.0	57.6	56.1	31.6	97.6	24.7	212	39.2	100	22.0	39.0
S40	48.8	33.6	119	66.9	85.8	58.6	58.2	32.2	93.9	24.4	187	39.5	93.3	21.1	39.7
S41	32.8	25.0	86.4	50.2	62.5	42.1	38.4	26.8	70.0	17.9	191	32.6	51.2	18.1	29.4
S42	44.6	30.6	112	63.0	79.1	53.2	51.0	31.4	89.8	21.2	214	37.7	78.4	20.5	36.7
S43	42.9	30.7	103	61.1	73.1	49.3	46.9	30.5	81.7	20.6	171	36.2	67.3	18.8	34.8
S44	44.4	30.3	108	62.5	76.8	52.6	49.5	31.5	87.3	21.5	170	37.3	60.9	19.6	36.2

S45	47.4	31.5	118	66.6	83.5	53.7	51.9	32.2	89.1	22.3	217	38.2	112	22.1	38.2
S46	45.9	30.3	106	61.6	76.3	53.7	51.8	30.5	91.1	23.6	237	36.0	93.6	21.7	37.2

[1] Alanine; [2] Glycine; [3] Valine; [4] Leucine; [5] Isoleucine; [6] Threonine; [7] Serine; [8] Proline; [9] Aspartate; [10] Methionine; [11] Glutamate; [12] Phenylalanine; [13] Lysine; [14] Histidine; [15] Tyrosine

Table S2. Fatty acid composition of *Spirulina* supplements available on the Slovenian market (% of total fatty acid content).

Sample	C14:0	C14:1	C15:0	C16:0	C16:1n7	C16:2n6	C16:2n4	C17:0	C17:1	C16:3n3	C18:0	C18:1n9c /C18:1n9t	C18:2n6c	C18:3n6	C18:3n3	C20:3n6	C22:0
S1	0.38	0.36	0.00	43.1	3.07	0.00	0.22	0.36	0.22	0.00	6.54	4.77	25.3	15.8	0.19	0.00	0.00
S2	0.52	1.10	0.00	40.9	5.58	0.00	0.00	0.39	0.55	0.00	10.6	8.35	19.1	12.8	0.35	0.00	0.00
S3	0.40	0.30	0.00	41.3	8.03	0.00	0.49	0.27	0.34	0.00	1.40	3.45	24.0	20.2	0.00	0.00	0.00
S4	0.37	0.46	0.00	42.0	7.58	0.00	0.42	0.25	0.45	0.00	2.64	5.24	21.0	20.1	0.00	0.00	0.00
S5	0.44	0.37	0.00	38.9	11.3	0.00	0.96	0.27	0.24	0.00	1.41	2.26	21.7	22.0	0.00	0.47	0.00
S6	0.59	0.00	0.22	18.4	5.56	9.71	9.38	0.35	0.00	9.58	4.13	4.36	22.0	0.00	18.4	0.00	0.27
S7	0.32	0.00	0.00	42.8	4.19	0.00	0.21	0.39	0.34	0.00	12.3	3.46	19.0	16.9	0.00	0.30	0.00
S8	0.36	0.45	0.00	41.7	5.85	0.00	0.39	0.37	0.31	0.00	4.52	3.66	21.5	20.5	0.00	0.24	0.00
S9	0.84	0.00	0.15	24.6	2.75	5.52	0.00	0.25	0.40	5.27	2.13	4.23	21.3	5.69	22.8	0.00	0.59
S10	0.29	0.12	0.00	42.1	7.88	0.00	0.51	0.27	0.31	0.00	1.71	3.55	22.3	21.0	0.00	0.00	0.00
S11	0.28	0.23	0.00	41.0	7.81	0.00	0.52	0.25	0.28	0.00	0.97	2.57	22.4	24.1	0.00	0.00	0.00
S12	0.33	0.33	0.00	42.6	6.66	0.00	0.41	0.35	0.36	0.00	1.76	3.74	22.7	20.9	0.00	0.00	0.00
S13	0.40	0.33	0.00	43.1	6.36	0.29	0.41	0.33	0.33	0.00	3.85	3.67	21.3	19.3	0.54	0.00	0.00
S14	0.35	0.14	0.00	41.4	5.49	0.00	0.40	0.27	0.32	0.00	9.56	2.52	21.1	18.5	0.00	0.00	0.00
S15	0.31	0.19	0.00	43.5	5.99	0.44	0.00	0.29	0.36	0.00	1.57	3.06	24.0	20.3	0.00	0.00	0.00
S16	0.36	0.00	0.00	44.8	6.25	0.00	0.37	0.00	0.31	0.00	1.98	4.33	21.9	19.7	0.00	0.00	0.00
S17	0.35	0.33	0.00	41.6	6.70	0.00	0.44	0.31	0.34	0.00	1.61	3.66	22.9	21.6	0.20	0.20	0.00
S18	0.24	0.08	0.00	28.3	4.15	14.6	0.20	0.12	0.23	1.56	0.88	3.66	34.4	8.84	2.52	0.00	0.00
S19	0.54	0.00	0.00	45.9	3.81	0.00	0.00	0.29	0.00	0.00	25.8	1.82	12.1	9.72	0.00	0.00	0.00
S20	0.35	0.41	0.00	42.1	6.94	0.00	0.44	0.33	0.35	0.00	4.32	4.01	20.8	19.7	0.00	0.24	0.00
S21	0.33	0.38	0.00	35.7	8.20	1.12	0.56	0.33	0.42	2.00	4.03	3.61	19.1	20.3	3.45	0.43	0.00
S22	0.41	0.33	0.00	45.1	4.44	0.42	0.00	0.33	0.29	0.35	12.1	3.88	17.9	13.8	0.70	0.00	0.00
S23	0.38	0.44	0.00	42.5	6.12	0.00	0.46	0.38	0.36	0.00	4.40	3.64	20.9	20.5	0.00	0.00	0.00
S24	0.34	0.41	0.00	44.7	6.54	0.00	0.44	0.40	0.41	0.00	2.47	4.88	20.7	18.6	0.00	0.00	0.00
S25	0.34	0.00	0.00	41.4	7.57	0.00	0.59	0.32	0.34	0.00	3.95	3.18	22.5	19.8	0.00	0.41	0.00
S26	0.40	1.12	0.00	38.8	7.96	0.00	0.39	0.42	0.72	0.00	3.12	9.34	22.5	14.6	0.67	0.00	0.00
S27	0.27	0.00	0.00	42.5	7.83	0.00	0.49	0.00	0.39	0.00	1.38	3.01	24.3	19.8	0.00	0.00	0.00
S28	0.30	0.00	0.00	43.3	6.41	0.00	0.38	0.27	0.35	0.00	5.09	3.93	22.2	17.9	0.00	0.00	0.00

S29	0.29	0.33	0.00	39.8	8.60	0.00	0.60	0.36	0.40	0.00	4.27	3.27	19.0	22.9	0.00	0.46	0.00
S30	0.28	0.34	0.00	40.3	8.69	0.00	0.55	0.33	0.37	0.00	4.49	3.25	18.8	22.3	0.00	0.42	0.00
S31	0.33	0.42	0.00	42.3	6.92	0.00	0.48	0.34	0.34	0.00	2.13	3.83	20.4	22.3	0.35	0.00	0.00
S32	0.37	0.42	0.21	41.6	7.27	0.00	0.44	0.28	0.34	0.00	4.19	4.03	20.9	19.9	0.00	0.00	0.00
S33	0.31	0.27	0.00	41.5	7.93	0.00	0.43	0.24	0.47	0.00	1.55	4.06	23.4	19.7	0.00	0.40	0.00
S34	0.63	0.69	0.00	39.8	8.14	0.21	0.48	0.37	0.31	0.00	7.50	3.00	19.9	17.3	0.45	0.72	0.00
S35	0.38	0.34	0.00	42.6	7.00	0.00	0.39	0.27	0.39	0.00	4.93	4.02	22.7	17.0	0.00	0.00	0.00
S36	0.50	0.77	0.00	42.1	8.01	0.00	0.48	0.37	0.37	0.00	1.92	4.55	22.4	18.5	0.00	0.00	0.00
S37	0.48	0.12	0.00	30.3	5.34	0.00	0.25	0.12	0.14	0.00	8.95	1.72	11.1	10.6	0.00	0.00	30.2
S38	0.31	0.00	0.00	40.6	4.87	0.00	0.26	0.40	0.50	0.00	9.88	4.27	22.3	16.4	0.00	0.29	0.00
S39	0.41	0.26	0.00	43.7	6.21	0.00	0.38	0.35	0.33	0.00	9.72	3.06	17.6	17.9	0.00	0.35	0.00
S40	0.34	0.00	0.00	41.0	5.23	0.00	0.27	0.39	0.51	0.00	10.3	4.43	22.1	15.3	0.00	0.30	0.00
S41	0.23	0.00	0.00	39.7	11.7	0.00	0.88	0.22	0.29	0.00	1.22	2.20	22.9	20.9	0.00	0.00	0.00
S42	0.51	0.53	0.00	43.9	8.37	0.00	0.49	0.33	0.44	0.00	1.66	4.43	22.5	16.8	0.00	0.35	0.00
S43	0.43	0.29	0.00	43.9	7.12	0.00	0.35	0.36	0.47	0.00	1.80	4.56	22.9	17.7	0.00	0.32	0.00
S44	0.31	0.80	0.00	38.7	9.13	0.00	0.41	0.27	0.64	0.00	1.25	7.66	23.5	16.5	0.00	0.82	0.00
S45	0.34	0.21	0.00	41.8	6.26	0.00	0.35	0.37	0.45	0.00	1.83	4.63	24.3	19.5	0.00	0.00	0.00
S46	0.26	0.00	0.00	40.6	6.99	0.00	0.38	0.00	0.41	0.00	1.69	4.00	31.0	14.5	0.00	0.34	0.00
